# Doping‐Induced Electronic/Ionic Engineering to Optimize the Redox Kinetics for Potassium Storage: A Case Study of Ni‐Doped CoSe_2_

**DOI:** 10.1002/advs.202200341

**Published:** 2022-04-25

**Authors:** Hui Shan, Jian Qin, Jingjing Wang, Hirbod Maleki Kheimeh Sari, Li Lei, Wei Xiao, Wenbin Li, Chong Xie, Huijuan Yang, Yangyang Luo, Gaini Zhang, Xifei Li

**Affiliations:** ^1^ Shaanxi international Joint Research Center of Surface Technology for Energy Storage Materials, Institute of Advanced Electrochemical Energy and School of Materials Science and Engineering Xi'an University of Technology Xi'an Shaanxi 710048 China

**Keywords:** diffusion dynamics, doping, electronic engineering, potassium‐ion batteries, transition metal selenides

## Abstract

Heteroatom doping effectively tunes the electronic conductivity of transition metal selenides (TMSs) with rapid K^+^ accessibility in potassium ion batteries (PIBs). Although considerable efforts are dedicated to investigating the relationship between the doping strategy and the resulting electrochemistry, the doping mechanisms, especially in view of the ion and electronic diffusion kinetics upon cycling, are seldom elucidated systematically. Herein, the crystal structure stability, charge/ion state, and bandgap of the active materials are found to be precisely modulated by favorable heteroatom doping, resulting in intrinsically fast kinetics of the electrode materials. Based on the combined mechanisms of intercalation and conversion reactions, electron and K^+^ ion transfer in Ni‐doped CoSe_2_ embedded in carbon nanocomposites (Ni‐CoSe_2_@NC) can be significantly enhanced via electronic engineering. Benefiting from the synthetic controlled Ni grains, the heterointerface formed by the intermediate products of electrochemical reactions in Ni‐CoSe_2_@NC strengthens the conversion kinetics and interdiffusion process, developing a low‐barrier mesophase with optimized potassium storage. Overall, an electronic tuning strategy can offer deeper atomic insights into the conversion reaction of TMSs in PIBs.

## Graphical abstract

Heteroatom doping has a significant impact on boosting the performance of secondary battery systems. By engineering the electrodes with controllable composites, ionic and electronic diffusion kinetics are simultaneously obtained. The underlying electrochemical K storage mechanisms based on the intercalation/deintercalation and conversion reactions are illustrated in detail by electrochemical kinetic analysis, theoretical calculations, and X‐ray absorption spectroscopy.

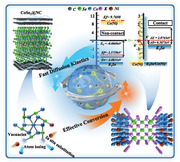

## 1 Introduction

In recent years, transition metal selenides (TMSs) have emerged as potential materials for potassium‐ion batteries (PIBs) owing to their abundant resources, diverse electronic properties, and high theoretical specific capacities. The electrochemical performance of TMSs is widely documented to be closely associated with the intrinsic ionic/electronic states and crystal structures.^[^ ^1^ ^]^ However, some inherent issues, including large volume expansion, sluggish ion transfer, and Jahn–Teller distortion (Co t^6^_2g_e^1^_g_) in TMSs, directly cause fast capacity fading and unfavorable rate capability.^[^ ^2^ ^]^ Extensive efforts,^[^ ^3^ ^]^ including multiple‐phase combination,^[^ ^4^ ^]^ anion‐cation doping,^[^ ^5^ ^]^ working voltage regulation,^[^ ^6^ ^]^ etc.,^[^ ^7^ ^]^ have been devoted to improving the reaction kinetics of the materials and achieving excellent performance.^[^ ^8^ ^]^ Notably, ion doping is defined as the time required to not only optimize the bandgap but also modulate the charge distribution in the doped TMSs, consequently enhancing the intrinsic conductivity and accelerating the reaction kinetics of the active materials for potassium ion storage.^[^ ^9^ ^]^ More significantly, heteroatom doping can adjust the van der Waals interactions and M—Se band via tunable interlayer spacing and charge distribution, leading to a stable process of K^+^ intercalation.^[^ ^10^, ^11^ ^]^ Controllable doping engineering also favors the reversible conversion reaction owing to the decreased potassium diffusion barriers.^[^ ^12^ ^]^ Additional density functional theory simulations proved a lower K^+^ migration energy for the doped samples during the potassiation process.^[^ ^13^ ^]^ However, of note, doping is not always constructive.^[^ ^14^ ^]^ Excess doping may distort the atomic structure and even lead to the degradation of the electrochemical performance of PIBs.^[^ ^15^ ^]^ Therefore, a specific doping strategy must be developed to obtain the optimum doping effect.^[^ ^16^ ^]^ To date, the impact of doping properties on potassium ion storage remains disputable and deserves careful investigation. Only few studies have been performed on the reconversion kinetics of doped TMSs as anodes for PIBs. Further, no review has sought to discuss the integrated impact of the reaction intermediates and doping on the electrochemical performance of PIBs.

In this study, a highly effective intercalation‐conversion reaction was investigated during potassium storage by transition‐metal doping. For Ni‐doped CoSe_2_ embedded in carbon nanocomposites (Ni‐CoSe_2_@NC), the specific effect of heteroatom doping on the local electronic state, atomic structure, and ionic diffusion kinetics was discussed in detail. Through electronic structure regulation, the potassium de/intercalation and reconversion kinetics were simultaneously enhanced owing to the stronger K^+^ adsorption and the developed heterointerface, including Ni grains. Consequently, the Ni‐CoSe_2_@NC has a considerable reversible capacity (400.7 mAh g^−1^ at 0.1 A g^−1^), which is 1.36‐fold greater than that of CoSe_2_@NC. In terms of conversion kinetics, this work presents an excellent doping guideline for developing advanced conversion‐type electrodes for PIBs with superior electrochemical performance.

## 2 Results and Discussion

**Figure 1:**
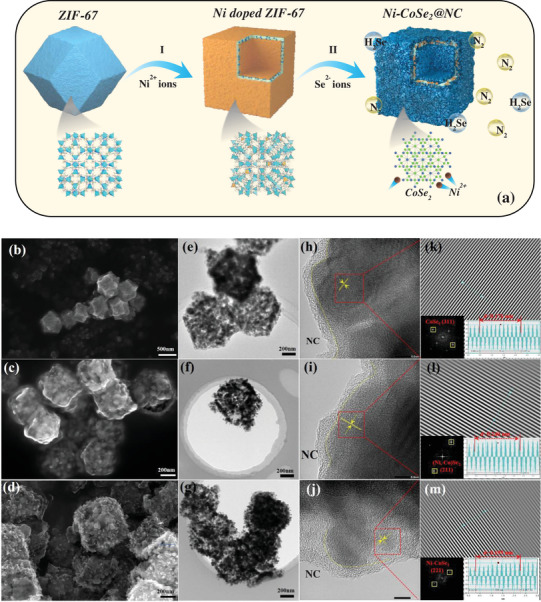
a) Schematic illustration for the fabrication of Ni‐CoSe_2_@NC. b–d) SEM, e–g) TEM, h–j) HRTEM, and k–m) inverse fast Fourier transformations (IFFT) of CoSe_2_@NC, (Ni, Co)Se_2_@NC, and Ni‐CoSe_2_@NC‐II. Insets are the corresponding FFT patterns and IFFT liner profiles.

The synthesis of Ni‐CoSe_2_@NC, as illustrated in **Figure** **1**a, can be divided into three stages. Briefly, the Co‐containing zeolitic imidazolate framework (ZIF‐67) with a rhombic dodecahedron structure (Figure S1, Supporting Information) was first fabricated using a typical coprecipitation process.^[^ ^17^ ^]^ Subsequently, the precursor with an average diameter of ≈2 *μ*m was treated with various amounts of Ni(NO_3_)_2_._6_H_2_O under moderate stirring to obtain Ni‐ZIF‐67 via a cation‐exchange reaction. In this etching process, protons derived from the hydrolysis of Ni^2+^ led to the release of Co^2+^ ions. Subsequently, Ni‐CoSe_2_@NC‐I/II/III and (Ni,Co)Se_2_@NC with incremental ratios of Ni to Co were obtained via the coprecipitation of Co^2+^/Ni^2+^, followed by ion exchange with Se^2−^ ions at 450 ℃ for 2 h. In this heating process, atomic vacancies appeared owing to the leaching of Co at the active sites of cube‐CoSe_2_. A sample with no Ni^2+^ ions (CoSe_2_@NC) was also prepared to serve as a reference; further details are provided in the Experimental Section.

The typical morphology and internal structure of the prepared products were detected by scanning electron microscopy (SEM) (Figure 1b–d) and transmission electron microscopy (TEM) (Figure 1e–g). After annealing, an inconspicuous morphological change occurred in the final samples. The confined nanoscale design was favored by the pyrolysis route of the MOFs, leading to a homogeneous distribution of CoSe_2_, Ni‐CoSe_2_, or (Ni,Co)Se_2_ nanoparticles embedded in the carbon matrix. The three typical samples with bold edges can be deduced to maintain a smaller dimension (≈600 nm) than ZIF‐67, which not only shortens the transfer paths for electrons/ions but also provides a large surface area for the active materials. Additional SEM images of the controllable‐doped samples are shown in Figure S2 in the Supporting Information. High‐resolution transmission electron microscopy (HRTEM) images (Figure 1h–j) also verified the same cubic structure of CoSe_2_@NC and Ni‐CoSe_2_@NC‐II. The lattice spacings of 1.760 Å for CoSe_2_@NC and 1.959 Å for Ni‐CoSe_2_@NC‐II corresponded to the (311) and (221) planes of trogtalite CoSe_2_. Regarding the (Ni, Co)Se_2_@NC nanocomposites, the lattice fringes of 2.40 Å matched well with the (211) plane of penroseite (Ni, Co)Se_2_ (Figure 1k–m). The elemental maps of Se, Ni, and Co in Figure S3 in the Supporting Information indicate a uniform elemental distribution and crystal dispersion.

**Figure 2:**
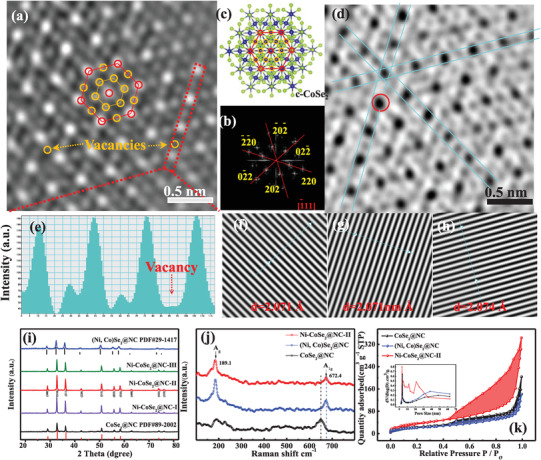
a) The HAADF‐STEM image with corresponding b) FFT and f–h) IFFT patterns of Ni‐CoSe_2_@NC‐II. c) Ball‐and‐stick model of the cubic‐CoSe_2_. d) ABF‐STEM image of Ni‐CoSe_2_@NC‐II. e) The intensity profiles along the selected rectangular regions suggest the missed surface Co atoms in Ni‐CoSe_2_@NC‐II. i) XRD patterns of CoSe_2_@NC, (Ni, Co)Se_2_@NC, and Ni‐CoSe_2_@NC‐I/II/III. j) Raman spectra and k) the nitrogen adsorption–desorption isotherms of CoSe_2_@NC, (Ni, Co)Se_2_@NC, and Ni‐CoSe_2_@NC‐II. The inset shows the corresponding pore size distribution curve.

High‐angle annular dark‐field scanning transmission electron microscopy (HAADF‐STEM) was used to assess the microstructure of Ni‐CoSe_2_@NC‐II. As shown in **Figure** **2**a, Ni‐CoSe_2_@NC‐II has a honeycomb structure at a higher magnification; however, the fast Fourier transformation (FFT) pattern (Figure 2b) and inverse FFT results (IFFT, Figure 2f–h) of Ni‐CoSe_2_@NC suggest an orientation along the [$\bar{1}$11] zone axis. The detailed IFFT profiles in Figure S4 in the Supporting Information indicate a lattice distance of 2.071 Å, which corresponds to the (220) plane. The ball‐and‐stick model of cube‐CoSe_2_ displayed in Figure 2c aligns well with the atomic resolution image and FFT simulation results (Figure S5, Supporting Information). The Co atoms are marked by orange circles, and the superposition of the Co and Se atoms is indicated by red circles. More importantly, some visible Co vacancies were readily distinctive in the HAADF‐STEM image and the corresponding intensity profile (Figure 2e) interpreted from the selected rectangular region, verifying the missing Co atoms after the doping process. The atomic dispersion in Ni‐CoSe_2_@NC‐II can be detected by annular bright field scanning transmission electron microscopy (ABF‐STEM). As displayed in Figure 2d, owing to the substitution of Ni for Co, some individual atomic arrangements appeared in an unorderly manner, which may form recombination centers, providing lateral proof for the specific site for the Ni atoms. The dispersive distribution of Ni in the final particles was also revealed by energy‐dispersive X‐ray spectroscopy (EDX) maps (Figure S6, Supporting Information).

X‐ray diffraction (XRD) was used to investigate the crystal structures of the final samples. As shown in Figure 2i, the XRD spectrum of the bimetallic selenide matched well with that of cubic‐(Ni, Co)Se_2_ (Joint Committee on Powder Diffraction Standards, JCPDS 29‐1417).^[^ ^18^ ^]^ The sharp peaks of Ni‐CoSe_2_@ NC‐I/II/III are indexed to cubic‐CoSe_2_ (JCPDS 89‐2002),^[^ ^19^ ^]^ which indicates the high purity of the final products and is consistent with the TEM results. Detailed lattice information of cubic CoSe_2_ is presented in Tables S1 and S2 in the Supporting Information. The relative contents of Co and Ni in the samples were estimated using inductively coupled plasma optical emission spectrometry. The results show that the atomic ratios of Ni: Co were ≈0.068, 0.100, 0.335, and 0.500 (Table S3, Supporting Information), which can be related to the experimental values of Ni‐CoSe_2_@NC‐I/II/III and (Ni, Co)Se_2_. Figure 2j shows the Raman spectra of CoSe_2_@NC, Ni‐CoSe_2_@NC‐II, and (Ni, Co)Se_2_@NC, in which the two peaks at ≈189 and 672 cm^−1^ are ascribed to the A_g_ and A_1g_ modes of CoSe_2_.^[^ ^20^ ^]^ Notably, the shift in the latter peak indicates some changes in the lattice symmetry induced by the incorporation of Ni atoms into CoSe_2_. Moreover, as shown in Figure S7 in the Supporting Information, the other characteristic peaks at 1582 and 1335 cm^−1^ are attributed to the G and D bands of the carbon framework.^[^ ^21^ ^]^ Indeed, the peak intensity ratio (I_D_/I_G_) differ in the obtained products, with Ni‐CoSe_2_@NC‐II exhibiting the largest value among them, indicating numerous defects in the nanoparticles. To confirm the porosity of the samples, N_2_ adsorption–desorption measurements were conducted. The Ni‐CoSe_2_@NC‐II nanocomposites displayed the largest Brunauer–Emmett–Teller surface area of 139.6 m^2^ g^−1^ (Figure 2k), suggesting that the doped Ni ions with catalytic and etching abilities toward carbon materials may be the reason for the formation of porous structures. The corresponding pore size distribution was analyzed using a nonlinear density functional model,^[^ ^22^, ^23^ ^]^ which reveal both meso‐ and micropore structures ranging from 1 to 25 nm (inset of Figure 2k). The carbon content of the samples was evaluated based on thermogravimetric analysis; more details are shown in Figure S8 in the Supporting Information.

**Figure 3:**
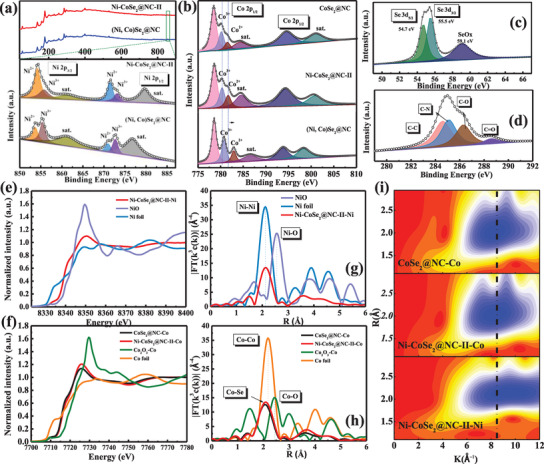
XPS spectra of the samples: a) Low resolution survey and high‐resolution Ni 2p of (Ni, Co)Se_2_@NC and Ni‐CoSe_2_@NC‐II; b) Co 2p of the three typical composites as well as c) Se 3d and d) C 1s on Ni‐CoSe_2_@NC‐II. e) Ni K‐edge XANES spectra and g) Ni K‐edge EXAFS spectra of Ni‐CoSe_2_@NC‐II. f) Co K‐edge XANES spectra and h) Co K‐edge EXAFS spectra of CoSe_2_@NC and Ni‐CoSe_2_@NC‐II. i) Wavelet transform (WT) contour plots of CoSe_2_@NC and Ni‐CoSe_2_@NC‐II.

The valence state and surface composition of the electrode materials were further studied by X‐ray photoelectron spectroscopy (XPS). The peaks associated with Co, Ni, Se, C, and O were observed in the full XPS spectra (**Figure** **3**a). Notably, in the high‐resolution Ni 2p spectrum, the peaks centered at 853.7 and 870.8 eV in Ni‐CoSe_2_@NC‐II and (Ni, Co)Se_2_@NC were well fitted by the Gaussian fitting method according to the 2p_3/2_ and 2p_1/2_ orbitals. Such finding suggests that most Ni ions in Ni‐CoSe_2_@NC‐II exist in the Ni^2+^ state, which may result in stable Ni−Se bonds and induce Co vacancies in the composites. More importantly, the ratio of the Ni^3+^to Ni^2+^ peak areas for (Ni,Co)Se_2_@NC (1.42) increased, implying that the reduction state of nickel increased with increasing Ni content in the synthetic process (Table S4, Supporting Information). Besides, two satellite peaks centered at 861.2 and 876.8 eV coincided well with the documented observations.^[^ ^24^ ^]^ The Co 2p spectrum in Figure 3b consists of two main peaks due to spin–orbit splitting, and each main peak is divided into two parts. As the Co atom is less electronegative, more electrons may be transferred to neighboring Ni atoms. Thus, the Co^3+^/Co^2+^ components in Co 2p_3/2_ of (Ni, Co)Se_2_@NC shifted to higher energies owing to more doped Ni ions and Co vacancies relative to Ni‐CoSe_2_@NC‐II, which may indicate the high oxidation of Co and further confirm the different electron distributions to neighboring Co atoms.^[^ ^25^ ^]^ The Se 3d peaks at 54.7 and 55.5 eV for Ni‐CoSe_2_@NC‐II are related to the Se 3d_5/2_ and Se 3d_3/2_ core levels, respectively, and the Se—O peak centered at 59.1 eV is due to the absorbed oxygen on the surface (Figure 3c).^[^ ^26^ ^]^ The relevant C 1s peak (Figure 3d) can be divided into four peaks that appear at 284.6, 285.15, 286.3, and 288.6 eV, and correspond to C—C, C—N, C—O, and C=O, respectively.^[^ ^27^ ^]^ The high‐resolution N 1s spectrum (Figure S9, Supporting Information) was associated with the pyridinic N, pyrrolic N, graphitic N, and oxidized N types.^[^ ^11^ ^]^ The active sites and conductivity of the electrodes can be increased by the N‐doping method, which is of great benefit to the electrochemical properties.^[^ ^28^ ^]^

The synchrotron radiation technology was used to elucidate the doping mechanism. As shown in Figure 3e, the Ni K‐edge X‐ray absorption near‐edge structure (XANES) spectra of Ni‐CoSe_2_@NC‐II are located between the NiO and Ni foil references, suggesting that the average valence of the Ni center is located between the oxidized states (Ni^2+^) and the reduced state (Ni^0^). These results may be due to unsaturated coordination at the Ni sites. Correspondingly, the Co K‐edge XANES spectra of CoSe_2_@NC and Ni‐CoSe_2_@NC‐II were found to be positioned in the middle of the referenced Co_2_O_3_ and Co foil, demonstrating that the average valence of Co was between Co^3+^ and Co^0^ (Figure 3f). Notably, the Co K‐edge for Ni‐CoSe_2_@NC‐II shifted to a higher energy than that of CoSe_2_@NC, which is not as evident from the XPS results and implies significant regulation of the electronic structure by the Ni doping strategy.^[^ ^29^ ^]^ Accordingly, some electrons may be transferred to the Ni^2+^ domains. Extended X‐ray absorption fine structure (EXAFS) spectroscopy was used to investigate the possible chemical coordination environment. For Ni‐CoSe_2_@NC‐II, the two main peaks of Ni—Se were identified at 2.45 and 3.83 Å while the Co—Se peaks were identified at 2.42 and 3.82 Å, respectively, as shown in Figure 3g,h. The corresponding fitting results are shown in Figure S10 and Tables S5 and S6 in the Supporting Information. At the atomic level, similar bond lengths confirmed the substitution of Ni for Co. The same Co—Se interactions in CoSe_2_@NC and Ni‐CoSe_2_@NC‐II suggest a stable local atomic environment in the samples after the doping process. Wavelet transforms (WT) were used to analyze the EXAFS oscillations of the samples.^[^ ^8^ ^]^ Figure 3i shows the WTs of the EXAFS oscillations for CoSe_2_@NC and Ni‐CoSe_2_@NC‐II. The intensities related to Co—Se and Ni—Se coordination for the two samples were found to be very close, suggesting that the local structure remains unchanged. The WT results further reveal the in situ substitution of Ni with Co atoms in the CoSe_2_ matrix.

To explore the effect of electronic structure engineering on electrochemical performance, the final products were employed as anodes in half‐cells versus K/K^+^.

**Figure 4:**
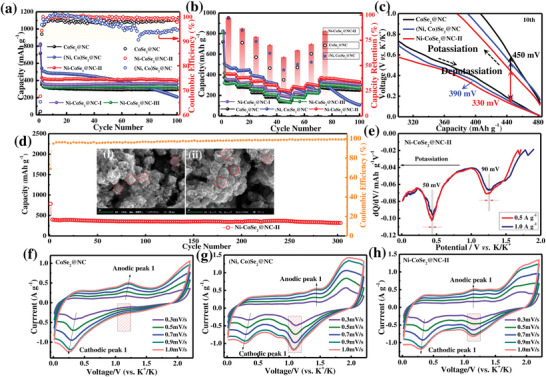
a) Cycling performance and coulombic efficiency at 0.1 A g^−1^ and b) rate performance and capacity retention at different current densities for the as‐prepared samples. c) Galvanostatic charge–discharge curves of CoSe_2_@NC, (Ni, Co)Se_2_@NC, and Ni‐CoSe_2_@NC‐II. d) Cycling stability at 0.2 A g^−1^ of the Ni‐CoSe_2_@NC‐II electrode and the insets are SEM images after 300 cycles. e) Differential capacity plots of the Ni‐CoSe_2_@NC‐II electrode at the selected current densities. f–h) CV curves of the CoSe_2_@NC, (Ni, Co)Se_2_@NC, and Ni‐CoSe_2_@NC‐II electrodes at different scan rates.

The cycling performance of all samples is presented in **Figure** **4**a. In contrast to CoSe_2_@NC, an appropriate amount of Ni substitution significantly improved the cyclability and reinforced the reversible capacity of Ni‐CoSe_2_@NC‐II. It can be found that the Ni‐CoSe_2_@NC‐II electrode delivers a specific capacity of 400.7 mAh g^−1^ at 0.1 A g^−1^ after 100 cycles, with a 36.0% increase relative to that of the original sample. Nevertheless, the initial Coulombic efficiency (ICE) of Ni‐CoSe_2_@NC‐II was only ≈65.39%. The relatively low ICE is due to the solid‐electrolyte interphase (SEI) consumption, which can be rapidly increased to 99.8% within ten cycles. The galvanostatic discharge/charge curves of Ni‐CoSe_2_@NC‐II are shown in Figure S11 in the Supporting Information, which is consistent with the reported TMS‐based anodes for PIBs.^[^ ^23^ ^]^ For (Ni, Co)Se_2_@NC, the specific capacity sharply declines to 208.2 mAh g^−1^ after 100 cycles at 0.1 A g^−1^ with a poor coulombic efficiency (95.1%). The results revealed that the electrochemical performance was successfully optimized by adjusting the atomic ratios of the two different cations. Figure 4b illustrates the rate capabilities of the samples ranging from 0.1 to 2.0 A g^−1^. Accordingly, the reversible capacities of Ni‐CoSe_2_@NC‐II decreased gradually and performed steadily at various current densities, ultimately outperforming those of CoSe_2_@NC, (Ni, Co)Se_2_@NC, and other controllable electrodes. Ni‐CoSe_2_@NC‐II delivered high average capacities of 406.4, 373.2, 337.6, 301.2, and 245.6 mAh g^−1^ at 0.1, 0.2, 0.5, 1.0, and 2.0 A g^−1^, respectively. Further, when the current returns to 0.1 A g^−1^, a comparative reversible capacity can quickly resume. Besides, Ni‐CoSe_2_@NC‐II delivered a steady capacity retention (59.97%) even at high current density (2.0 A g^−1^), which is superior to CoSe_2_@NC (36.80%) and (Ni, Co)Se_2_@NC (46.94%). The outstanding rate performance of the modified electrode is primarily attributed to the electronic engineering toward the rational structure, doping elements, and induced defects, which intrinsically enhance its electrochemical activity. In particular, as shown in Figure 4c, the Ni‐CoSe_2_@NC‐II electrode exhibited a lower overpotential (330 mV) than CoSe_2_@NC (450 mV) and bimetallic selenide (Ni, Co)Se_2_@NC (390 mV), indicating facilitated K^+^ diffusion kinetics.^[^ ^30^ ^]^ In a long‐term cycling test at 0.2 A g^−1^ (Figure 4d), the Ni‐CoSe_2_@NC‐II electrode could still maintain a reversible capacity of 320 mAh g^−1^ after 300 cycles, with a low capacity fading of ≈0.3% per cycle (relative to the second cycle). According to SEM analysis (insets in Figure 4d), the cubic morphology was preserved after 300 cycles, confirming the structural stability and high robustness of Ni‐CoSe_2_@NC‐II. To obtain vital insights into the electrochemical kinetics of the electrodes, d*Q*/d*V* curves were plotted at current densities of 0.5 and 1 A g^−1^. As depicted in Figure 4e, Ni‐CoSe_2_@NC‐II had small potential gaps of ≈50 and ≈90 mV for the specific peaks, whereas those for the (Ni, Co)Se_2_@NC electrode were ≈150 and 125 mV, respectively (Figure S12, Supporting Information), which further elucidates the optimized K^+^ transport in the doping materials.^[^ ^31^ ^]^ To investigate the K^+^ storage mechanism in PIBs, additional electrochemical analyses and computational simulations were performed. First, during the initial cyclic voltammetry (CV) profiles recorded at a scan rate of 0.1 mV s^−1^, as shown in Figure S13 in the Supporting Information, the irreversible peak close to 1.0 V for all three samples can be attributed to the SEI film formed at the electrolyte‐electrode interface during discharge. Using Ni‐CoSe_2_@NC‐II as an example, the two reduction peaks observed at ≈1.02 and 0.44 V are associated with the K^+^ intercalation process (Ni‐K*_x_* CoSe_2_) and conversion process (K_2_Se and Ni‐Co). Conversely, the oxidation peaks at 1.07 and 1.94 V correspond to the reverse conversion reaction and the deintercalation process. To evaluate the ion transport kinetics of the electrodes, the domination of pseudocapacitive behavior was analyzed using different CV curves from 0.3 to 1.0 mV s^−1^ (Figure 4f–h). CoSe_2_@NC is observed to proceed with no intercalation behavior during the potassiation process, while the K^+^ intercalation delivers a significant difference due to Ni doping of Ni‐CoSe_2_@NC‐II and (Ni, Co)Se_2_@NC. Subsequently, with the increased K^+^ intercalation, the M—Se bonds (M = Co, Ni) in K*_x_* MSe_2_ are broken, suggesting the start of conversion reactions toward potassium. More information on the pseudo‐capacitance behaviors of K^+^ storage is presented in Figure S14 in the Supporting Information.

**Figure 5:**
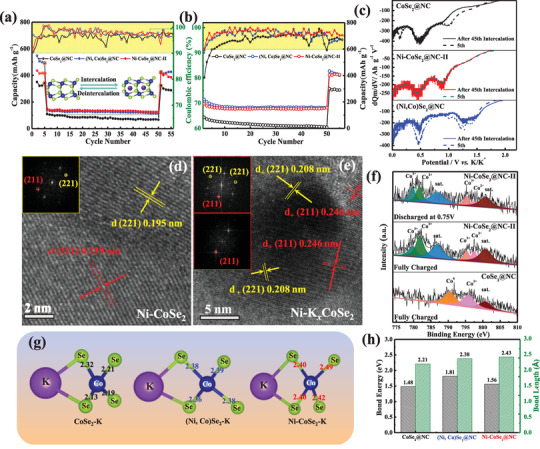
a) Cycling performance of CoSe_2_@NC, Ni‐CoSe_2_@NC‐II, and (Ni, Co)Se_2_@NC under three stages of voltage windows: 0.01–2.2 to 0.75–2.2 V, then 0.01–2.2 V. b) Cycling performance of CoSe_2_@NC, Ni‐CoSe_2_@NC‐II, and (Ni, Co)Se_2_@NC under two stages of voltage windows: 0.75–2.2 V then 0.01–2.2 V based on two mechanisms of intercalation and conversion. c) Differential capacity plots of CoSe_2_@NC, Ni‐CoSe_2_@NC‐II, and (Ni, Co)Se_2_@NC in 0.01–2.2 V before and after 45 cycles of K^+^ de/intercalations. HRTEM images and corresponding FFT patterns of Ni‐CoSe_2_@NC‐II at different discharge–charge stages in PIBs: d) fully charged to 2.2 V and e) discharged to 0.75 V. f) High‐resolution XPS spectra of Ni‐CoSe_2_@NC‐II collected at different charged states and CoSe_2_@NC in Co 2p region. g) The main Se−Co bonds formed in the interfaces and their corresponding bond lengths and bond energy are shown in (h).

To further elaborate the doping effect on the intercalation reaction, the cycle performances of CoSe_2_@NC, Ni‐CoSe_2_@NC‐II, and (Ni, Co)Se_2_@NC were evaluated in three‐stage potential windows. Under a wider voltage window (0.01–2.2 V), the electrodes demonstrated higher reversible capacities owing to the mixed conversion and de/intercalation reactions. When the voltage range was shortened to 0.75–2.2 V, only intercalation‐induced reactions occur. Further, when the voltage range was changed from 0.01–2.20 to 0.75–2.20 V, Ni‐CoSe_2_@NC‐II displayed a more stable intercalation‐induced charge capacity of ≈130 mAh g^−1^ than the bare CoSe_2_@NC electrode (see **Figure** **5**a), which then recovers to a reversible capacity of ≈420 mAh g^−1^ in the voltage range of 0.01–2.20 V, aligning with the results presented in Figure 4a. Besides, if the cycling performance test begins with the potential window of 0.75–2.20 V, the intercalation‐induced charge capacity of CoSe_2_@NC rapidly decreased to the lowest value after 50 cycles and the capacities returned to ≈400 mAh g^−1^ in subsequent cycles with the voltage range of 0.01–2.20 V, as shown in Figure 5b. In contrast, Ni‐CoSe_2_@NC‐II and (Ni, Co)Se_2_@NC exhibit ultrafast K^+^ intercalation with superior coulombic efficiencies. Accordingly, the differential capacity (d*Q*/d*V*) plots suggest that the intercalation reaction preferentially occurred in Ni‐CoSe_2_@NC‐II and (Ni, Co)Se_2_@NC (Figure 5c). The disappearance of the peak at ≈1.0 V for CoSe_2_@NC after the 45th intercalation proves that the doping strategy can stabilize the intercalation reaction and simultaneously boost the conversion reaction. The corresponding CV curves are consistent with the results of the differential capacity plots shown in Figure S16 in the Supporting Information. The relevant charge/discharge cycles are displayed in Figure S17 in the Supporting Information and the changes in peak values in the d*Q*/d*V* curves for the CoSe_2_@NC, Ni‐CoSe_2_@NC‐II, and (Ni, Co)Se_2_@NC electrodes during the intercalation process for a voltage range of 0.75–2.20 V are visualized in Figure S18 in the Supporting Information. The structural evolution and chemical state at different charge–discharge stages were further characterized by HRTEM and XPS. In the fully charged state (2.2 V), lattice fringes of Ni‐CoSe_2_@NC‐II were observed with interplanar spacings of 0.195 and 0.239 nm, indexed to the (221) and (211) planes of cubic CoSe_2_ (JCPDS 89‐2002) as shown in Figure 5d. After K^+^ intercalation (at 0.75 V), the d‐spacings of the (221) and (211) planes extended to 0.208 and 0.246 nm, respectively (Figure 5e), without any phase change. The corresponding IFFT profiles are shown in Figure S19 in the Supporting Information, and more relevant HRTEM images are shown in Figure S20 in the Supporting Information. The XPS spectrum of Co_2p_ in CoSe_2_@NC revealed the formation of a new peak in the fully charged state corresponding to the Co^0^ metal; this formation can be attributed to the poor reversibility and sluggish kinetics during the electrochemical process (Figure 5f). As a result, the effect of Ni doping on the chemical bonds was further investigated by first‐principles calculations. The bond lengths and bond energies of the samples after large K^+^ insertion are shown in Figure 5g,h. The average Se—Co bond energy in CoSe_2_@NC was 1.48 eV, but was strengthened to 1.56 eV through the doping methods with Ni‐CoSe_2_@NC‐II. An appropriate M—Se bond energy can stabilize the intercalation reaction; however, an obsessively high bonding energy hinders the formation and breaking of the M—Se bonds, ultimately affecting the conversion reaction. The potassium intercalation behavior benefits from the longest bond length. The lack of an intercalation peak at ≈1.02 V for CoSe_2_@NC provides evidence for this perspective. The weak bond energy in the materials may not be powerful enough to push the intercalation process; however, Ni doping thermodynamically allows K ions to be more easily intercalated into CoSe_2_.

**Figure 6:**
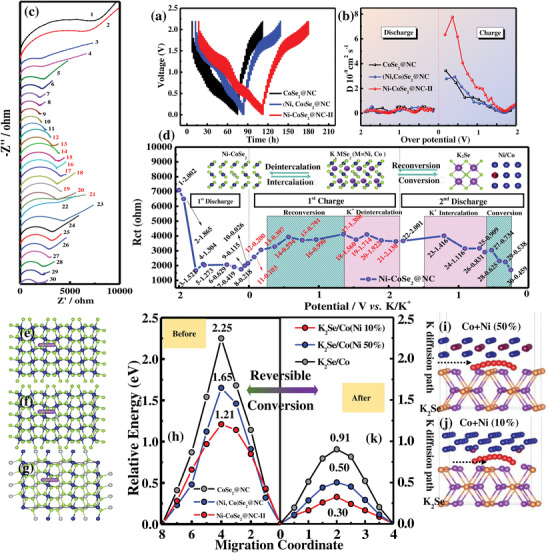
a) GITT voltage profiles and b) the corresponding K^+^ diffusion coefficients of CoSe_2_@NC, (Ni, Co)Se_2_@NC, and Ni‐CoSe_2_@NC‐II in the 2nd cycle. c) Nyquist plots of Ni‐CoSe_2_@NC‐II collected at various discharge/charge depths in the frequency range of 100 kHz–10 mHz. d) The dependencies in fitting parameters of *R*_ct_ on discharge potential for the Ni‐CoSe_2_@NC‐II electrode. e–g) K diffusion paths in the ideal structure of CoSe_2_, Ni‐CoSe_2_, and (Ni, Co)Se_2_. i,j) The K_2_Se/Co(Ni) interface for (Ni, Co)Se_2_ and Ni‐CoSe. h,k) The corresponding K^+^ diffusion energy barriers during the reversible depotassiation/potassiation process in the electrode materials.

The galvanostatic intermittent titration technique (GITT) further validated the enhanced K^+^ diffusion ($D_{K}^{+}$) rate in the doped materials, which is a vital parameter for achieving excellent rate capability in PIBs. The electrodes were activated at 0.1 A g^−1^ to obtain steady SEI films before performing the GITT measurement. A typical single step of the GITT curves is displayed in Figure S21 in the Supporting Information, and the $D_{K}^{+}$ can be calculated using equation^[^ ^32^ ^]^

(1) $$D_{K^{+}}=\frac{4}{\pi \tau }{\left(\frac{m_{B}V_{M}}{M_{B}A}\right)}^{2}{\left(\frac{\Delta E_{S}}{\Delta E_{\tau }}\right)}^{2}$$

where *τ* is the time of the current pulse, *A* and *m*_B_ */M*_B_ are the area and mass/molar mass of the electroactive material, respectively, and *V*_M_ is the molar volume of the electrode. Regardless of the IR drop, Δ*E*_S_ and Δ*E_*τ*_* denote the voltage change in the steady state through the current pulse and the voltage difference before and after the current pulse, respectively. **Figure** **6**a shows the voltage responses of the CoSe_2_@NC, (Ni, Co)Se_2_@NC, and Ni‐CoSe_2_@NC‐II electrodes during the GITT test. Additionally, the corresponding diffusion coefficients were calculated, as shown in Figure 6b. Accordingly, the average diffusion coefficient ($D_{K}^{+}$) of Ni‐CoSe_2_@NC‐II was higher than that of the other two samples, which can be attributed to the controllable Ni‐doping strategy. Of note, $D_{K}^{+}$ steepened at the start of the depotassiation process, especially for Ni‐CoSe_2_@NC‐II, suggesting that the discharging products have a positive effect on the conversion reaction and markedly boost ion diffusion in the electrochemical process.^[^ ^33^ ^]^ The electrochemical impedance spectra (EIS) were examined (Figure S22a, Supporting Information). Based on a comparison, the lower semi‐infinite diffusion resistance and charge‐transfer resistance of Ni‐CoSe_2_@NC‐II suggest kinetically profitable electrochemical behavior, which agrees well with the GITT and CV results.^[^ ^34^ ^]^ Figure 6c and Figure S23 in the Supporting Information show the EIS spectra of the Ni‐CoSe_2_@NC‐II electrode at various discharge/charge depths during the first and a half cycle. Based on the equivalent circuit (Figure S22b, Supporting Information),^[^ ^35^ ^]^ the *R*_ct_ values initially declined at ≈1.52 V, and then were steady from 1.30 to 0.03 V, demonstrating the stable properties of the SEI layer in the first discharge process and the function of metallic Co(Ni) nanoparticles with superior conductivities (Figure 6d).^[^ ^36^ ^]^ Subsequently, in the first charge process, the *R*_ct_ values gradually increased upon the reconversion reaction and remained essentially constant during the continuous potassium ion deintercalation, indicating that the electrochemical reaction could be more difficult under a high potential without boosting the K_2_Se/Co(Ni) heterointerface.^[^ ^37^ ^]^ Subsequently, in the second discharge process, the *R*_ct_ values remained stable during the K^+^ intercalation process (≈1.02 V). When discharging the conversion reaction toward K_2_Se and Co/Ni grains (≈0.44 V), the *R*_ct_ values decreased with the formation of the heterointerface, further confirming the value of $D_{K}^{+}$ calculated based on the GITT results (Figure 6b). Therefore, the *R*_ct_ values can be concluded to be maintained under K^+^ de/intercalation and change periodically during re‐/conversion reactions. The corresponding conversion routes of the modified electrodes during cycling are summarized in the insets of Figure 6d.

**Figure 7:**
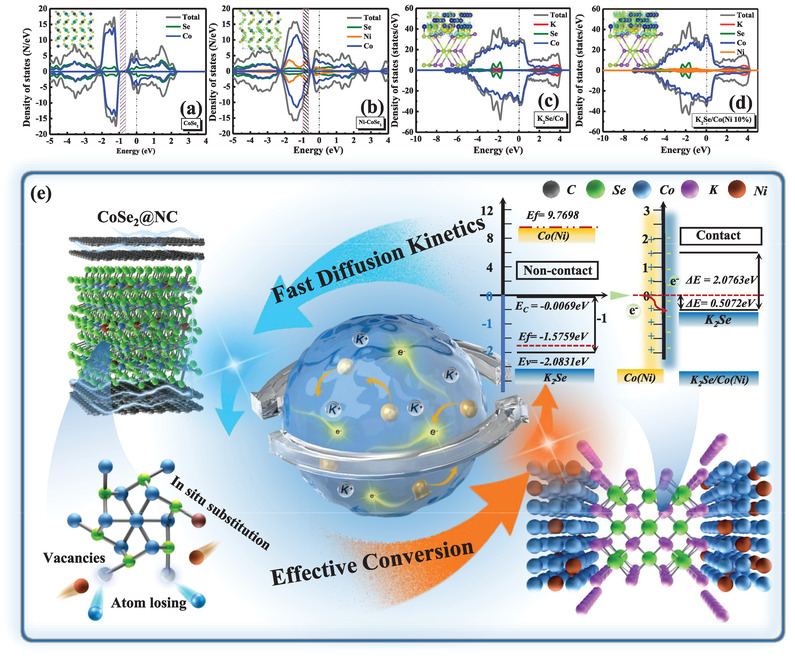
a–d) Calculated density of states (DOS) analyses of CoSe_2,_ Ni‐CoSe_2,_ K_2_Se/Co as well as K_2_Se/Co(Ni) heterointerfaces. Insets are the corresponding charge density difference, respectively. e) Schematic illustration of enhanced electrochemical kinetics of Ni‐CoSe_2_@NC‐II electrode.

Theoretical computations were performed to investigate the electrochemical reaction mechanisms in detail.^[^ ^37^ ^]^ Rate capability is well known to be significantly influenced by the level of ion mobility. A lower barrier leads to a more rapid migration of ions through the bulk phase.^[^ ^38^ ^]^ The K atom migration paths in the three typical models are shown in Figure 6e–g. Ni‐CoSe_2_ was found to deliver a conspicuously lower K diffusion barrier (1.21 eV) than CoSe_2_ (2.25 eV) and (Ni, Co)Se_2_ (1.65 eV) (Figure 6h). After the conversion reaction, a new interface between K_2_Se and Co(Ni) developed under fully discharged conditions, and the K^+^ diffusion barriers decreased significantly (Figure 6k).^[^ ^29^, ^39^ ^]^ Hence, the vital role of the heterointerface was confirmed and the corresponding potassium migration pathways are shown in Figure 6i,j. More importantly, the electrochemical properties of the intermediate heterointerface in the doped material (0.33 eV) were recognized to be superior to those of bimetallic TMSs (0.50 eV), which demonstrates the importance of accurately controllable doping methods. The density of states (DOS) of bulk CoSe_2_ and Ni‐CoSe_2_ were compared. At ≈−0.5–1.0 eV, the number of electronic states for Ni‐CoSe_2_ was higher than that of CoSe_2_, verifying the enhanced electrical conductivity of the nanocomposites after applying the doping strategy (**Figure** **7**a,b). After the conversion reaction, the naturally developed K_2_Se/Co and K_2_Se/Co(Ni 10%) heterointerfaces exhibited metallic properties, as shown in Figure 7c,d. Owing to the incorporation of Ni atoms, the heterointerface results in an optimized effect on the reshaping of the electronic structure of the electrode materials, suggesting a more conductive characteristic of the K_2_Se/Co(Ni 10%).^[^ ^40^ ^]^ The calculated DOS analysis and corresponding charge density difference of the K_2_Se/Co(Ni 50%) interface are shown in Figure S24 in the Supporting Information. Conclusively, the controllable electronic engineering not only facilitates the potassium intercalation, but also drives the conversion reaction during the electrochemical process, ultimately leading to an enhancement in the K^+^ storage capacity. Furthermore, the charge density differences were analyzed to gain more insights into the impact of doping on the samples. As depicted in the corresponding insets, the Ni‐CoSe_2_ interface showed more electron accumulation, implying reinforced charge transfer. To provide an atomistic evaluation, the interfacial behavior was investigated during the operation. As shown in the insets of Figure 7e, the Fermi level of the Co(Ni) grains was evidently higher than that of K_2_Se, while shifting toward lower potentials upon contact. Thus, positive charges accumulate on the Co(Ni) side and negative charges on the K_2_Se side, leading to the development of an in‐built electric field pointing from Co(Ni) to K_2_Se.

The superiorities relative to the counterparts of Ni‐CoSe_2_ can be summarized as follows: i) Ni doping effectively functioned to optimize the electronic states of CoSe_2_, markedly enhancing the intrinsic conductivity; high potassium storage could also be achieved. ii) Doping induced stronger bonding energy and higher K^+^ adsorption effectively drove the potassium intercalation reaction, which assists in high redox activity. iii) Owing to the developed heterointerface of K_2_Se/Co(Ni), the built‐in field significantly boosted electronic transport. A favorable diffusion energy barrier is beneficial for the fast conversion reaction kinetics during the discharge/charge process. Overall, this study confirms the high efficiency of the obtained Ni‐CoSe_2_@NC electrodes for PIBs and proposes a novel approach for the preparation of advanced materials by optimal doping engineering in energy storage systems.

## 3 Conclusion

In summary, we proposed an approach to regulate the electronic structure of TMSs and revealed its effect on the energy storage mechanisms of Ni‐CoSe_2_@NC for PIBs. By precisely controlling the Ni:Co ratio, the doped samples exhibited improved electrochemical properties within simultaneously developed ionic and electronic diffusion. The enhanced intercalation‐conversion kinetics of CoSe_2_‐based anodes can be effectively operated by electronic engineering. As a result, the modified sample exhibits improved potassium storage with a high reversible capacity of 400.7 mAh g^−1^ after 100 cycles (98% retention), and a superior rate capability of 284.0 mAh g^−1^ at 2 A g^−1^. Overall, this study provides deep insights and a unique approach that favors intercalation and conversion reactions by heteroatom doping of TMSs, which not only alters the traditional definition toward the reaction mechanism but also provides a novel idea for the design of high‐performance conversion‐type anodes for PIBs.

## Conflict of Interest

The authors declare no conflict of interest.

## Supporting information

Supporting Information

## Data Availability

The data that support the findings of this study are available from the corresponding author upon reasonable request.
