# Supplementary material for: Doping‐Induced Electronic/Ionic Engineering to Optimize the Redox Kinetics for Potassium Storage: A Case Study of Ni‐Doped CoSe2
Source: Adv Sci (Weinh). 2022 Apr 25;9(18):2200341. doi: 10.1002/advs.202200341 (PMC9218747; doi:10.1002/advs.202200341)
Supplement: Supplementary file 1 — Supporting Information [file ADVS-9-2200341-s001.pdf]

ADVANCED SCIENCE
Open Access

Supporting Information

for *Adv. Sci.*, DOI 10.1002/advs.202200341

Doping-Induced Electronic/Ionic Engineering to Optimize the Redox Kinetics for Potassium Storage: A Case Study of Ni-Doped $CoSe_2$

Hui Shan, Jian Qin, Jingjing Wang, Hirbod Maleki Kheimeh Sari, Li Lei, Wei Xiao, Wenbin Li, Chong Xie, Huijuan Yang, Yangyang Luo, Gaini Zhang and Xifei Li**

Supporting Information

Doping-induced Electronic/Ionic Engineering to Optimize the Redox Kinetics for Potassium Storage: A Case Study of Ni-doped $CoSe_2$

Hui Shan, Jian Qin, Jingjing Wang, Hirbod Maleki Kheimeh Sari, Li Lei, Wei Xiao, Wenbin Li, Chong Xie, Huijuan Yang, Yangyang Luo, Gaini Zhang, Xifei Li**

H. Shan, Prof. J. Qin, HMK. Sari, W. Liu, Y. Hao, X. Song, L, Wang, J. Wang, C. Xie, W.Xiao, Prof. X. Li
Institute of Advanced Electrochemical Energy & School of Materials Science and Engineering, Xi'an University of Technology
Shaanxi international Joint Research Center of Surface Technology for Energy Storage Materials
Xi'an, shaanxi, 710048, china
E-mail: qinjian@xaut.edu.cn; xfli@xaut.edu.cn

Experimental Section

Preparation of ZIF-67: All chemical reagents were of analytical grade and directly used without further purification. Typically, 0.328 g of 2-methylimidazole (Sigma-Aldrich) was dissolved in 25 mL of methanol. Subsequently, 0.328 g of $Co(NO_3)_2$•$6H_2O$ was added into the clear solution. After vigorous stirring for 30 min, it was incubated at room temperature for 24 h. The obtained precipitate was collected by centrifugation, repeatedly washed with methanol at 6000 rpm for at least 3 times, and then dried in a vacuum oven at 80 °C overnight to obtain purple ZIF-67 crystals.

Preparation of Ni-ZIF-67 precursors: ZIF-67 (40 mg) was dispersed into absolute ethanol (20 ml) to from a homogeneous purple dispersion. Then, add 10-60 mg $Ni(NO_3)_2 \cdot 6H_2O$ solution (dissolved in 5 ml absolute ethanol) was slowly added into the former dispersion. After stirring for 1h under room temperature, the product of Ni-ZIF-67 was washed, filtrated, and dried with the same process.

Preparation of Ni doping $CoSe_2$@NC: Commercial selenium powder (50 mg) was mixed with the prepared Ni-Zif-67 precursors (25 mg), annealing at 450 °C for 90 min with temperature increasing rate of 2 °C min^{-1}. By tuning the ratios of Ni to Co sources in the composites, the corresponding products were denoted as Ni-$CoSe_2$@NC-I/II/III and (Ni, Co)Se_2@NC, respectively. For the synthesis of $CoSe_2$@NC, it was prepared with the same process as that of Ni-$CoSe_2$@NC but without the addition of $Ni(NO_3)_2 \cdot 6H_2O$ sources.

Materials Characterization: XRD (*/SmartLab), SEM (ZEISS, MERLIN Compact) TEM (JEOL JEM-2100Plus) and HADDF scanning transmission electron microscopy

(JEOL JEM-ARM200CF) were applied to analyze the crystalline phase, morphology, and nanostructure of the obtained samples. The contents of Ni, Co and Se were determined by inductively coupled plasma (ICP). Raman spectroscopy (HR Evolution) and XPS (Thermo Fisher) were carried out to investigate the surface characteristics. Thermogravimetric analysis (TGA, Perkin Pyris Diamond) was conducted with a temperature ramp of 10 °C min^{-1}. Brunauer–Emmett–Teller (BET) surface area and nitrogen adsorption isotherm were measured over P/Po = 0-1 at 77 K on an autosorb IQ (ASAP 2020 Plus HD88). The X-ray absorption spectra of Co *K*-edge and Ni *K*-edge were conducted in fluorescence mode at the BL14W1 beamline of the Shanghai Synchrotron Radiation Facility, China, operated at 3.5 GeV with maximum injection currents of 230 mA. The synchrotron beam was monochromatized using a double-crystal monochromator equipped with a Si (111) crystal to reduce the harmonic component of the monochrome beam. The Co foil, Ni foil, Co_2O_3 and NiO standards were used as reference sample.

Electrochemical Measurements: To fabricate the anode materials of PIBs, CMC, super carbon (SP) and active materials were weighed with the ratio of 1:2:7; then the obtained homogeneous slurry was uniformly painted on a copper foil and dried for 12 h at 60 °C. The foil was cut into wafers with area of 1.13 cm^2, taken as the electrodes. The active mass loading on the circular pieces was about 1 mg. The electrolyte was 1.0 M KSFI in diethyl carbonate (DEC) and ethylene carbonate (EC) (1:1 in volume). Potassium metal was used as counter electrodes and glass microfiber membranes were served as separators. The electrochemical performance was conducted in the potential

of 0.01–2.20 V on BTS TestControl. Cyclic voltammetry (CV) and impedance spectroscopy (EIS) were performed on CHI660E (CHI electrochemistry workstation).

Theoretical calculation: The Spin-polarization density functional theory (DFT) calculations within the generalized gradient approximation (GGA) was performed using the Perdew-Burke-Ernzerhof (PBE) formulation.[1] The projected augmented wave (PAW) potentials were chosen to describe the ionic cores and valence electrons were taken into account using a plane wave basis set with a kinetic energy cutoff of 450eV.[2] Partial occupancies of the Kohn−Sham orbitals were allowed using the Gaussian smearing method and a width of 0.05 eV. The electronic energy was considered self-consistent when the energy change was smaller than 10−6 eV. A geometry optimization was considered convergent when the energy change was smaller than 0.03 eV $Å^{-1}$. What's more, K ions migration barrier energies were evaluated using the climbing nudged elastic band (CI-NEB) methods.

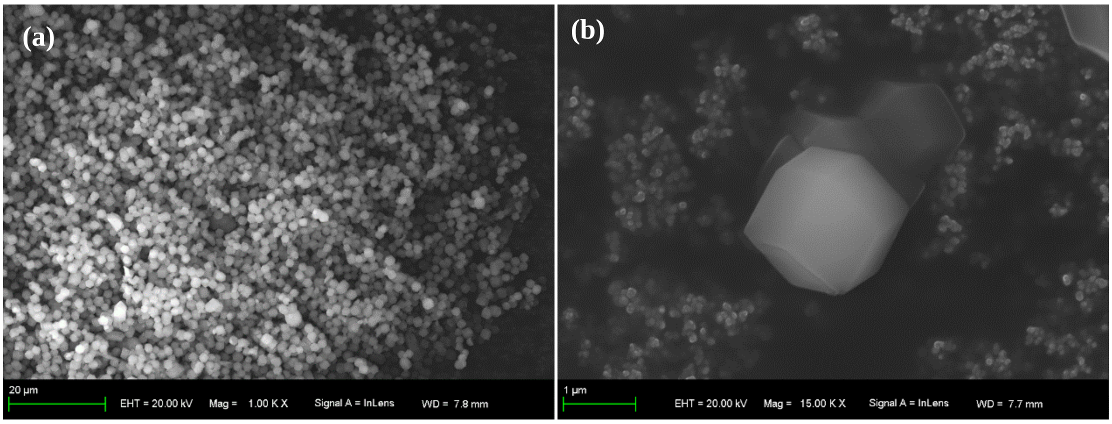

Figure S1. SEM images of ZIF-67.

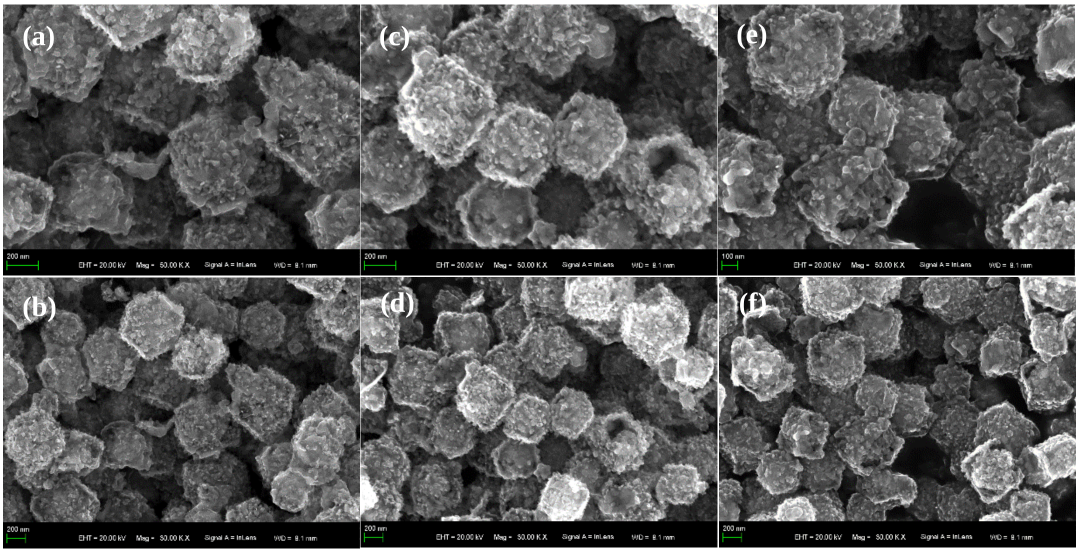

Figure S2. SEM images of Ni-$CoSe_2$@NC-I(a,b), -II(c,d), and -III(e,f).

The morphologies and structures of Ni-$CoSe_2$@NC-I/II/III can be investigated at different magnification. As shown in Figure S2, the high-magnification SEM images display the surface of the nanospheres are covered with many fine particles and become coarse. By using ZIF-67 as the template, Ni-$CoSe_2$@NC nanocomposites are successfully formed.

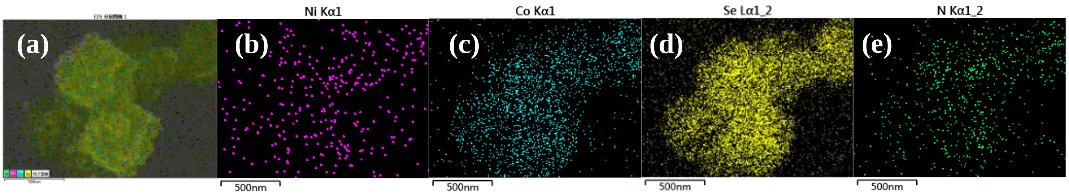

Figure S3. Elemental mapping images of (Ni,Co)Se_2@NC.

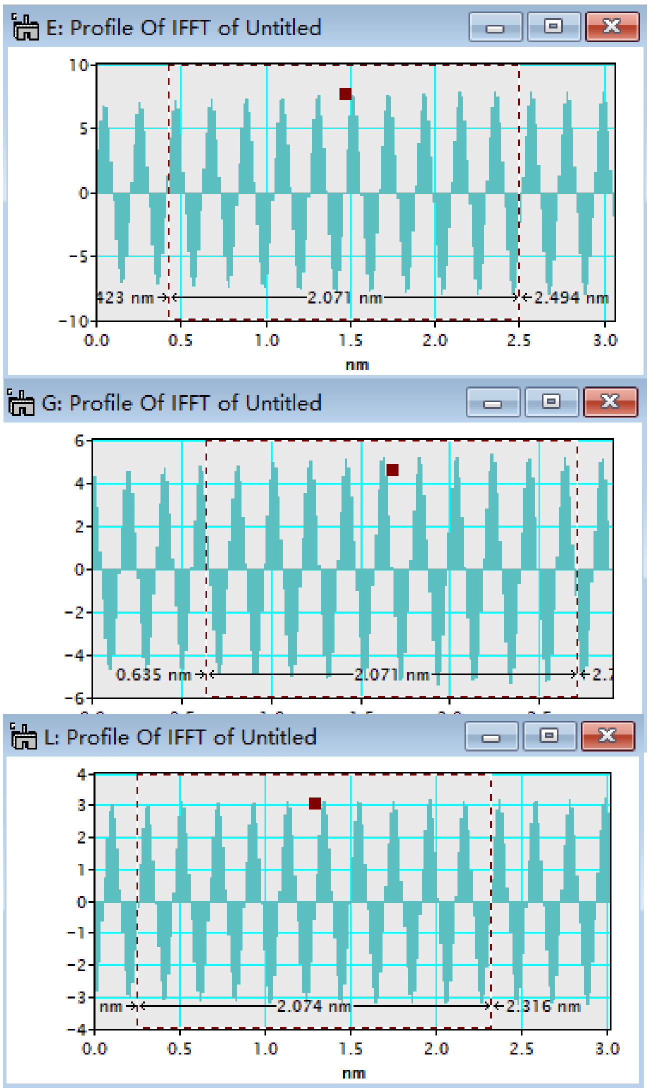

Figure S4. Inverse FFT liner profiles of Ni-$CoSe_2$@NC-II.

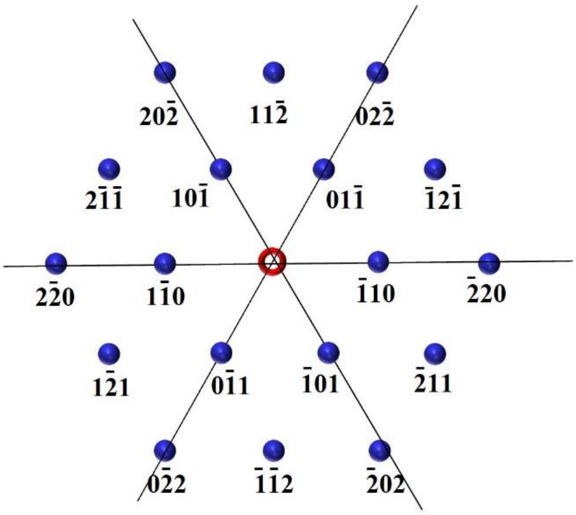

Figure S5. Simulated FFT pattern of the c-$CoSe_2$ (zone axis: [111]).

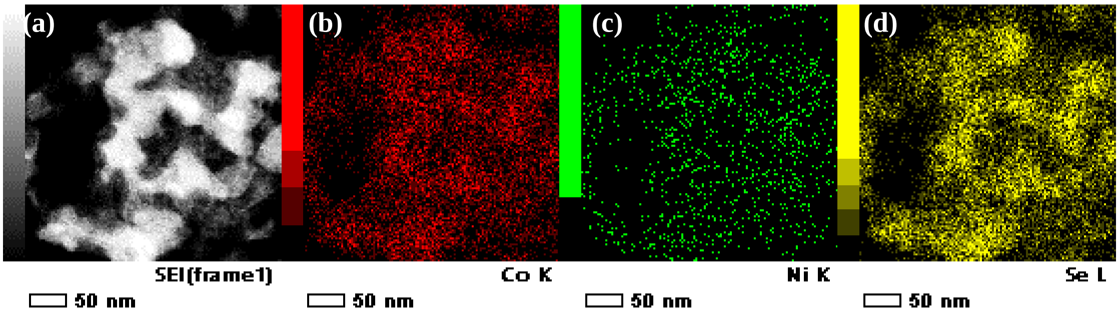

Figure S6. The dispersive X - ray spectroscopy (EDX) maps of Ni-$CoSe_2$@NC-II.

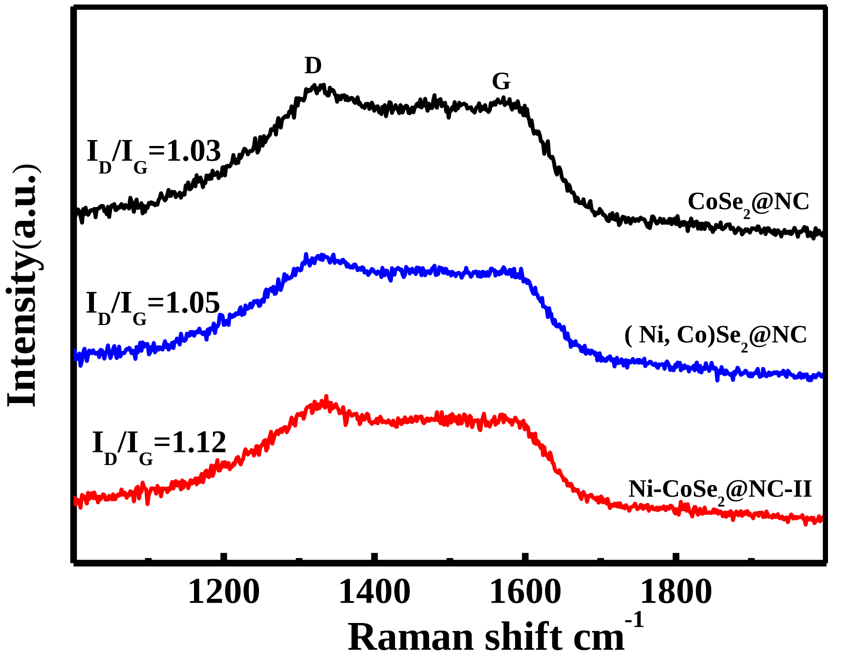

Figure S7. Raman spectra of $CoSe_2$@NC, Ni-$CoSe_2$@NC-II and (Ni,Co)Se_2@NC.

The D-band reveals the structural defects, and the G-band corresponds to sp^2-hybridized graphitic carbon. The high ID/IG ratio in the samples suggests the existence of defects in the carbon matrix.[3]

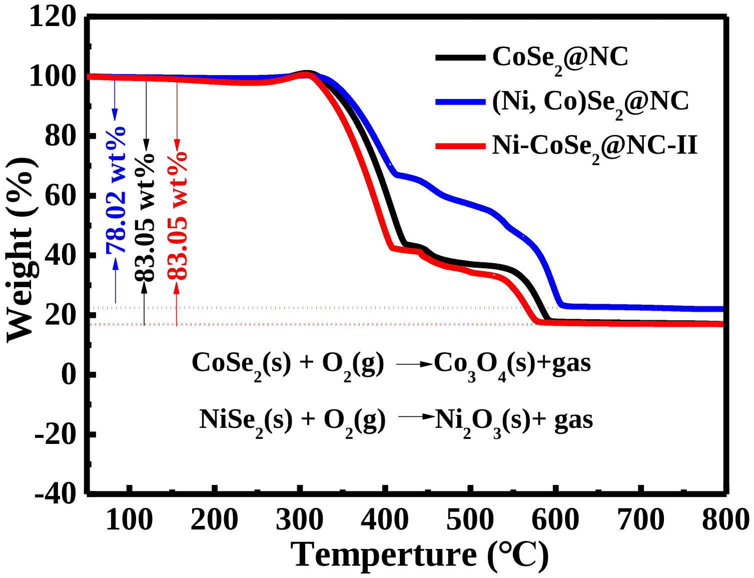

Figure S8. TGA curves of $CoSe_2$@NC, Ni-$CoSe_2$@NC-II and (Ni, Co)Se_2@NC.

The calculation of carbon content in the composite: The weight loss below 200 °C was assigned to the loss of absorbed water. The weight loss above 800 °C can be corresponded to the generation of Co_3O_4, Ni_2O_3 and the combustion of carbon.

Cure A

For $CoSe_2$@NC:

We assume that the weight of $FeSe_2$ is x%. Based on the equations:

$$3CoSe_2@NC \rightarrow Co_3O_4 + gas$$

Atomic weight: Co (59), Se (79), O (16), Ni (58.7≈59)

$$\frac{241x}{217*3} = 16.95\% \qquad x = 45.79\%$$

Cure B

The content (wt.%) ratio of Ni: Co is 0.5 for (Ni, Co)Se_2@NC by ICP.

We assume that the weight of $CoSe_2$ is x, so the the weight of $NiSe_2$ is about 0.5 x,

$$2NiSe_2@NC \rightarrow Ni_2O_3 + gas$$

$$\frac{241x}{217*3} + \frac{165.4x}{216.7*2} * 0.5 = 1 - 78.02\%$$

$$x = 39.18\%$$

$$C(w\%) = 41.23\%$$

Cure C

The content (wt.%) ratio of Ni: Co is 0.1 for Ni-$CoSe_2$@NC-II by ICP.

We assume that the weight of $CoSe_2$ is x, so the the weight of $NiSe_2$ is about 0.1 x,

$$2NiSe_2@NC \rightarrow Ni_2O_3 + gas$$

$$\frac{241x}{217*3} + \frac{165.4x}{216.7*2}*0.1 = 1 - 83.05\%$$

$$x = 41.51\%$$

$$C(w\%) = 54.34\%$$

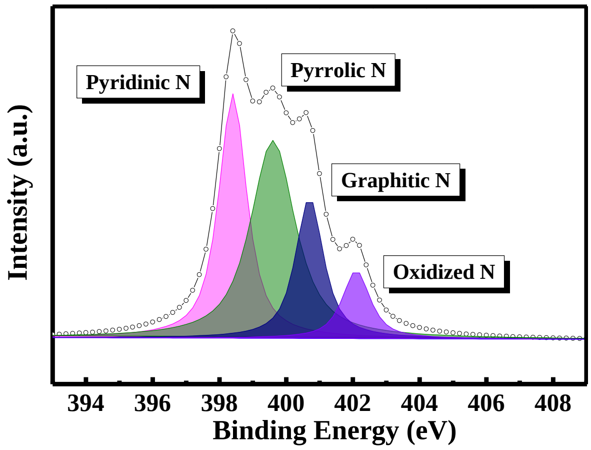

Figure S9. N 1s spectra of Ni-$CoSe_2$@NC-II.

Using deconvolution method, the N 1s spectrum was fitted by the assumption of four species: pyridinc N (398.4), pyrrolic N (399.6 eV), graphitic N (400.9 eV) and oxidized N (402.1 eV).[4]

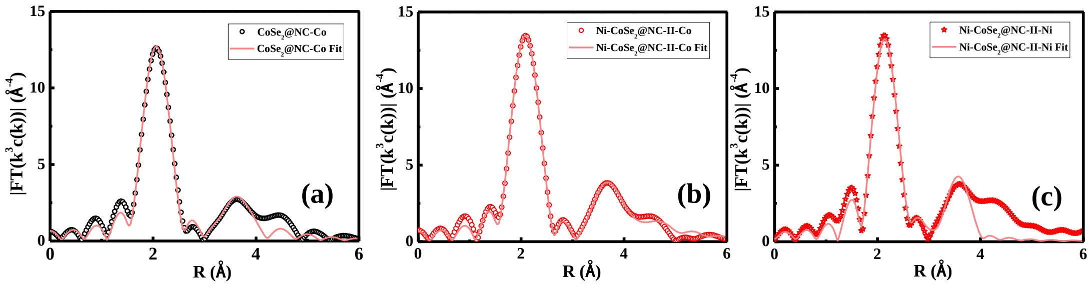

Figure S10. The EXAFS fitting results of the Co K-edge for $CoSe_2$@NC and Ni-$CoSe_2$@NC-II as well as the Ni K-edge for Ni-$CoSe_2$@NC-II.

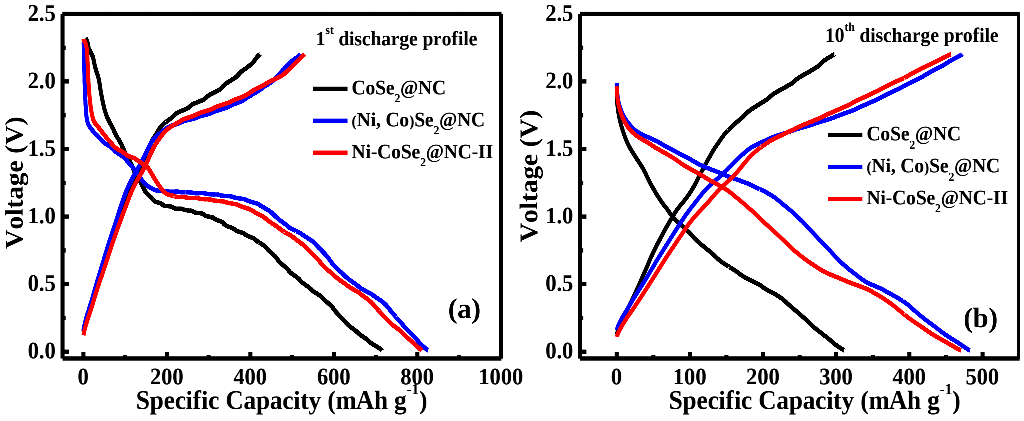

Figure S11. a) The 1st and b) 10th galvanostatic charge-discharge curves of $CoSe_2$@NC, Ni-$CoSe_2$@NC-II and (Ni, Co)Se_2@NC.

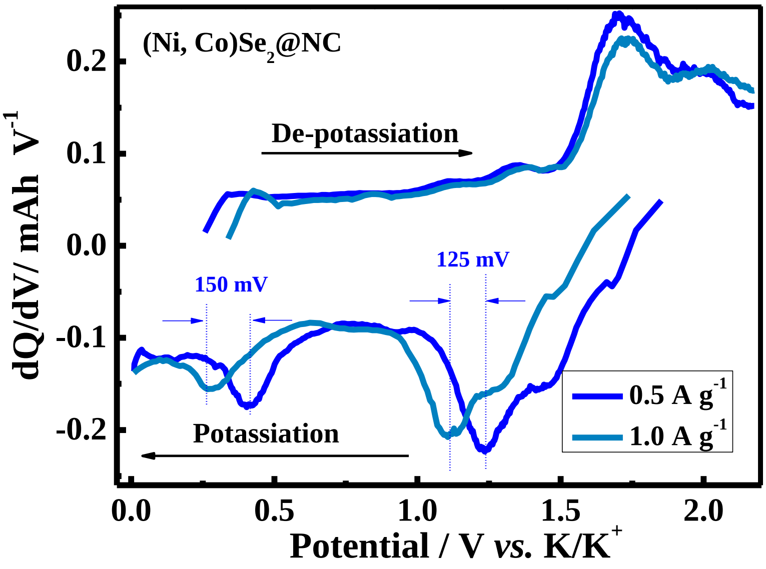

Figure S12. Differential capacity plots of (Ni, Co)Se_2@NC electrode at the selected current densities.

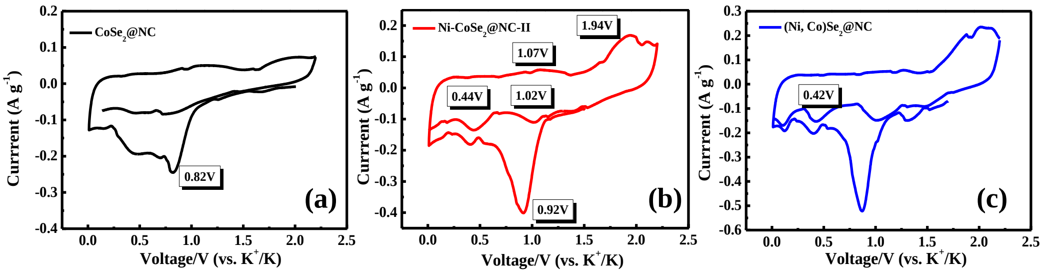

Figure S13. CV curves at a scan rate of 0.1 mV s^{-1} of $CoSe_2$@NC, Ni-$CoSe_2$@NC-II and (Ni, Co)Se_2@NC.

The relevant chemical reaction equations are as following:

Stage I (1.07V), reconversion:

$$Co + 2K_2Se \rightarrow K_XCoSe_2 + (4-x)k^+ + (4-x)e^-$$

Stage II (1.94V), K^+-deintercalation:

$$K_XCoSe_2 \rightarrow CoSe_2 + xk^+ + xe^-$$

Stage III (1.02V), K^+-intercalation:

$$CoSe_2 + xk^+ + xe^- \rightarrow K_xCoSe_2$$

Stage IV (0.44V), conversion:

$$K_xCoSe_2 + (4-x)k^+ + (4-x)e^- \rightarrow Co + 2K_2Se$$

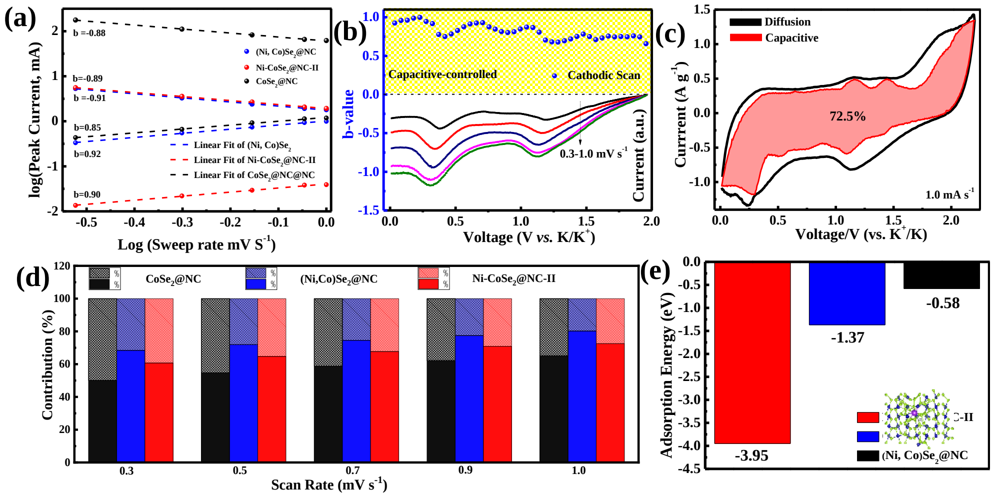

Figure S14. a) Log (i) versus Log (v) plots at peak currents corresponding to the CV curves. b) b-values calculated through cathodic scan for the Ni-$CoSe_2$@NC-II electrode. c) The shaded region shows the CV profile of Ni-$CoSe_2$@NC-II with pseudocapacitive contribution at a scan rate of 1.0 mV s^{-1}. d) Capacitive-controlled contribution at different scan rates and e) the relavent calculations of K^+ adsorption energy for $CoSe_2$@NC, (Ni, Co)Se_2@NC and Ni-$CoSe_2$@NC-II.

Concerning the contribution based on the two major mechanisms of pseudo-capacitance and K^+ insertion/extraction, the peak current (i) and the scan rate (v) follow the equations: [5]

$$i = av^b\#(1)$$

$$logi = k_1logv + loga\#(2)$$

In which, a is a constant coefficient and, b is calculated based on the slope of log i versus log υ. The electrochemical reaction is dominated by the surface capacitive behavior (b value closes to 1.0) or diffusion-controlled intercalation behavior (b value closes to 0.5).[6] After fitting, the b values of $CoSe_2$@NC, Ni-$CoSe_2$@NC-II and (Ni, Co)Se_2@NC in PIBs can be calculated to be -0.80/0.85, -0.89/0.90 and -0.91/0.90 in the anodic and cathodic processes at different scan rates, as displayed in Figure S14a, which accordingly, both samples exhibit capacitive-dominant characteristics.[7] Taking the Ni-$CoSe_2$@NC-II anode as an instance, b-values resulted from the voltage range of 2.2-0.01 V in the discharge process are shown in Figure S14b. Notably, all the b-values are nearly 0.75 during the whole cathodic scan, demonstrating an almost linear relationship between the scan rate and current.[8] Furthermore, it reveals a capacitive ratio of 72.5% at a specific scan rate of 1.0 mV s^{-1} (Figure S14c). The high contribution of the surface-driven process can be ascribed to the well-controlled structure with an appropriate doping content of Ni ions. The detailed proportions of capacity dominated by the capacitive behavior are summarized in Figure S14d and Table S7. Additionally, the models of equilibrium states for the three typical samples with K atom are optimized (Figure S15) and the corresponding adsorption energy of K for each model is illustrated in Figure S 14e, which is calculated to be -0.58, -1.73 and -3.95 eV. It further confirms that Ni-$CoSe_2$@NC-II is constructive for the adsorption of K^+, thereby boosting the K^+ intercalation kinetics.[9] Hence, the reaction

mechanisms on the K^+ intercalation/deintercalation should be investigated by regulating the potential window.

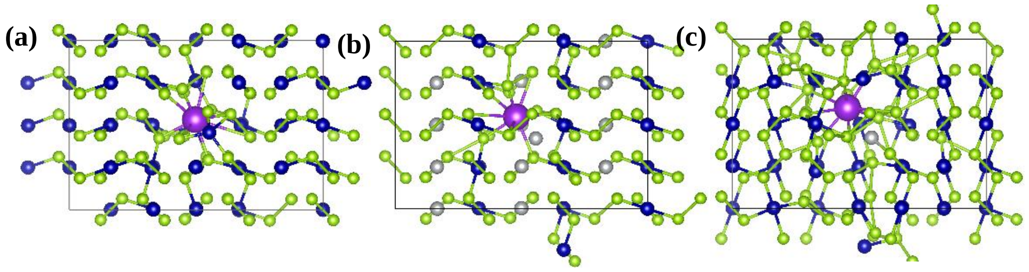

Figure S15. The optimized models of equilibrium states with one K atom for a) $CoSe_2$@NC, b) (Ni, Co)Se_2@NC and c) Ni-$CoSe_2$@NC-II.

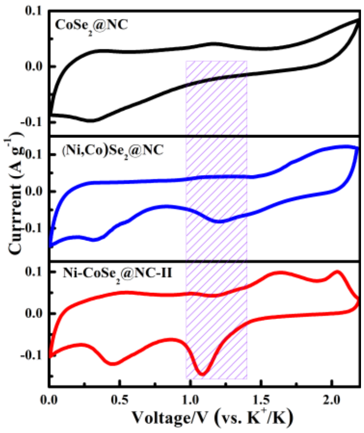

Figure S16. CV curves of $CoSe_2$@NC, (Ni, Co)Se_2@NC and Ni-$CoSe_2$@NC-II.

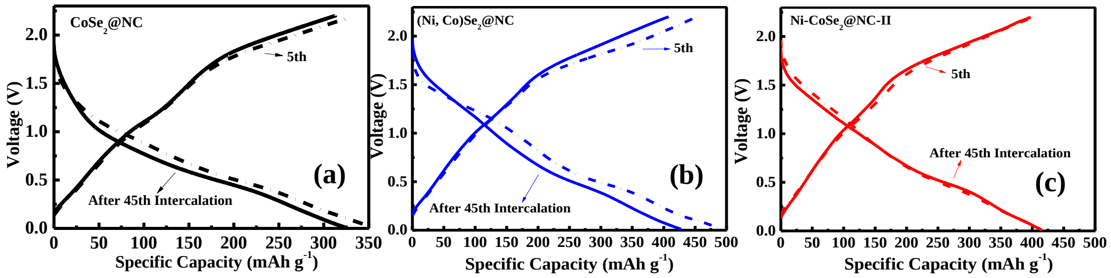

Figure S17. Galvanostatic charge-discharge curves of a) $CoSe_2$@NC, b) Ni-$CoSe_2$@NC-II and c) (Ni, Co)Se_2@NC in 0.01-2.2 V before and after 45 cycles of K^+ de/-intercalations.

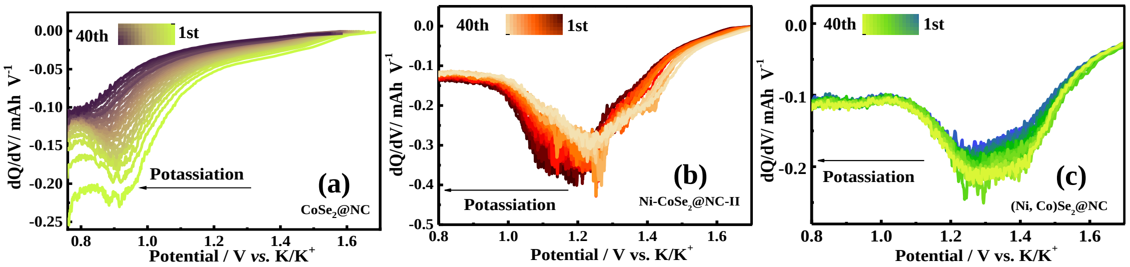

Figure S18. Differential capacity plots of a) $CoSe_2$@NC, b) Ni-$CoSe_2$@NC-II and c) (Ni, Co)Se_2@NC in 0.75-2.2 V.

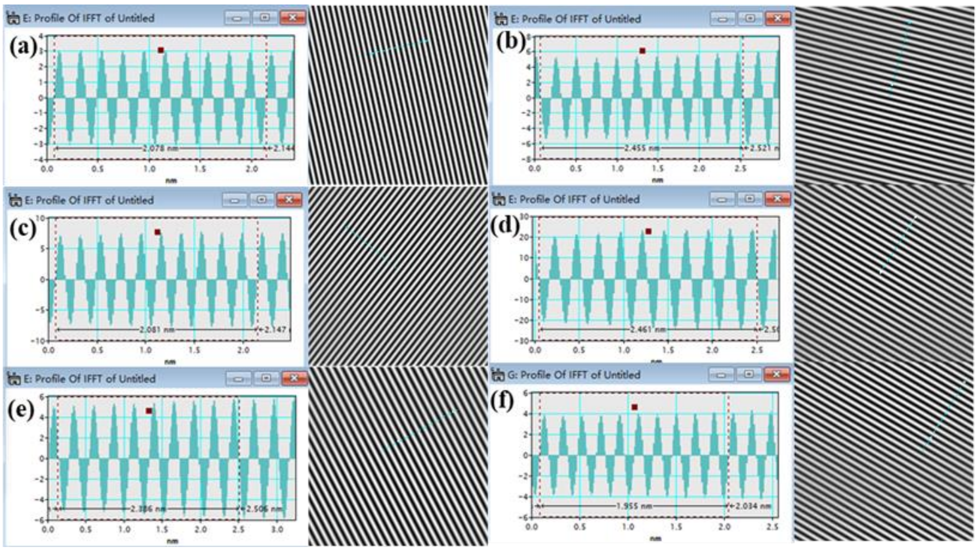

Figure S19. Inverse FFT liner profiles of Ni-$CoSe_2$@NC-II in PIBs: a-d) when dishcarged to 0.75V and e,f) fully charged to 2.2V.

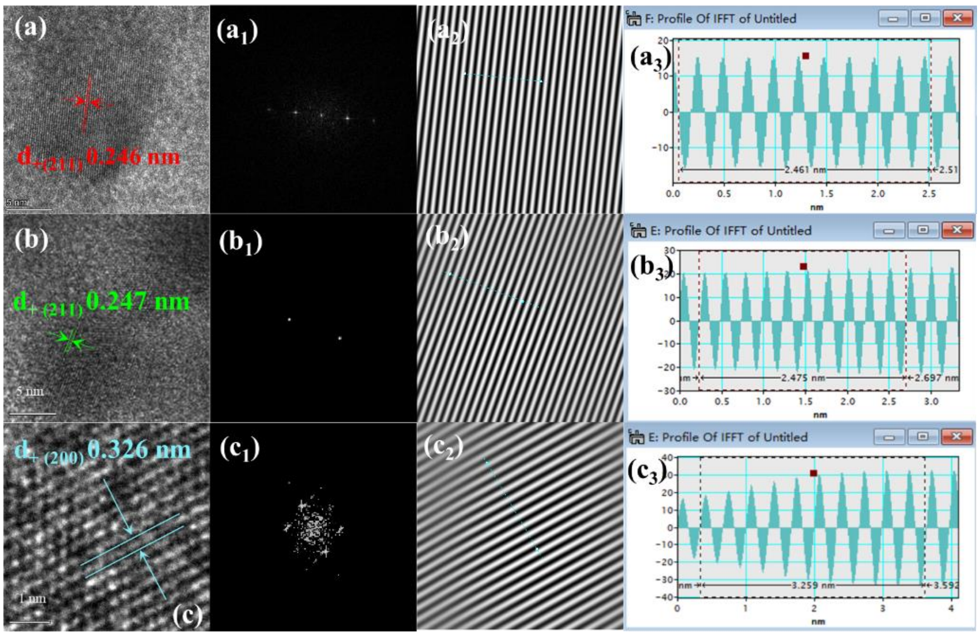

Figure S20. More HRTEM analyses of the Ni-$CoSe_2$@NC-II electrode when discharged to 0.75V.

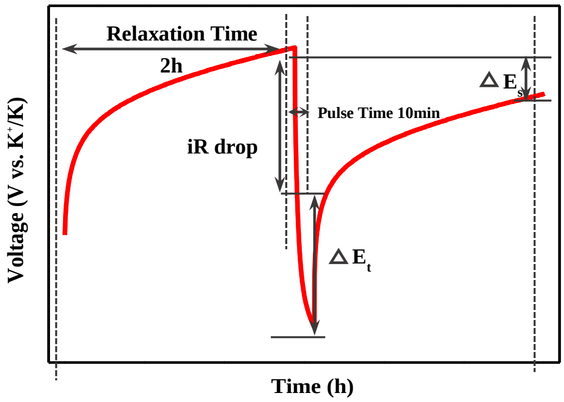

Figure S21. E vs. t curves of Ni-$CoSe_2$@NC-II electrode in a single GITT.

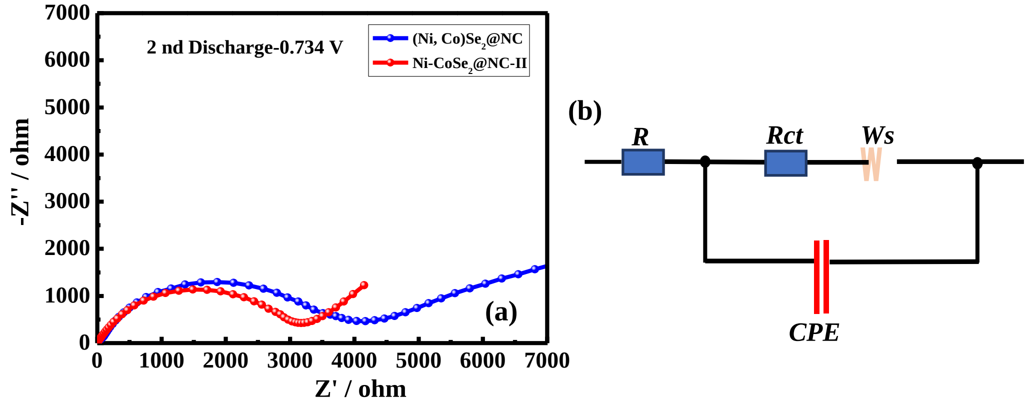

Figure S22. EIS spectra of Ni-$CoSe_2$@NC-II and (Ni, Co)Se_2@NC electrodes at 0.734 V in the 2nd discharge process.

In the equivalent circuits, *Rs* is the total resistance of the electrolyte, electrode, current collector and separator. *Rct* is sum of the interface resistance from SEI and the charge transfer resistance. *Ws* is the Warburg impedance, and *CPE* stands for constant phase element, which is related to the capacitive behavior during the respective process.[10] Notably, the curves are composed of a nearly straight line in the low-frequency region and a depressed semicircle in the high frequency range, representing the Warburg impedance related to the solid-state diffusion and the charge-transfer impedance (Rct), respectively.

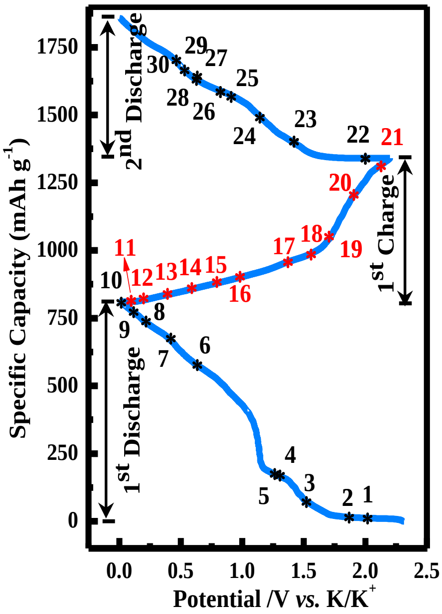

Figure S23. Discharg-charge curves of the Ni-$CoSe_2$@NC-II electrode: each point represents an EIS measurement.

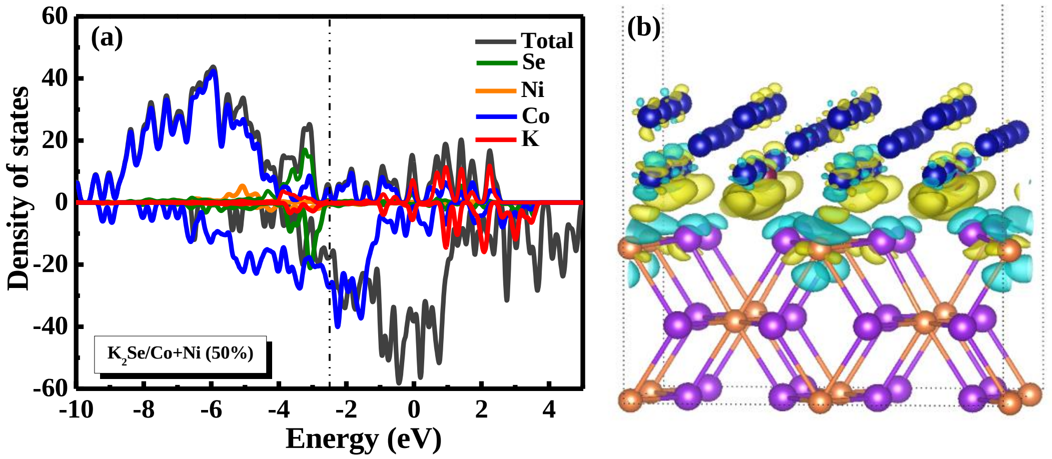

Figure S24. a) Calculated density of states (DOS) analysis and b) corresponding charge density difference of the K_2Se/Co(Ni 50%) interface.

It can be seen that this heterointerface has continuous distributions around the Fermi level, indicating that the K_2Se/Co(Ni 50%) also has the metallic properties.[1] Nevertheless, compared with that of K_2Se/Co(Ni 10%), there were less electron accumulation (yellow color) in the K_2Se/Co(Ni 50%) interface, which indicated that K_2Se/Co(Ni 10%) has high electronic conductivity duo to the rational doping content.

Table S1. Detailed lattice parameters (cell) of the cubic $CoSe_2$.

Chemical formula	Cell‹a*b*c› / Phase‹ α * β * γ ›	a	b	c
$CoSe_2$ 89-2002	Cubic ‹90.0*90.0*90.0›	5.8593	5.8593	5.8593

Table S2. $CoSe_2$ with the cubic phase (JCPDS 89-2002).

2-Theta	d(Å)	I(f)	(h k l)	Theta	1/(2d)	2pi/d
26.323	3.3829	0.8	(1 1 1)	13.162	0.1478	1.8574
30.487	2.9297	27.9	(2 0 0)	15.244	0.1707	2.1447
34.19	2.6204	100	(2 1 0)	17.095	0.1908	2.3978
37.57	2.3921	78.2	(2 1 1)	18.785	0.209	2.6267
43.657	2.0716	13.2	(2 2 0)	21.829	0.2414	3.033
46.456	1.9531	0.2	(2 2 1)	23.228	0.256	3.217
51.699	1.7666	51	(3 1 1)	25.85	0.283	3.5566
54.182	1.6914	3.8	(2 2 2)	27.091	0.2956	3.7147
56.588	1.6251	21.4	(0 2 3)	28.294	0.3077	3.8664
58.93	1.566	31.6	(3 2 1)	29.465	0.3193	4.0124
63.451	1.4648	4.9	(4 0 0)	31.726	0.3413	4.2894
65.645	1.4211	0.2	(4 1 0)	32.822	0.3518	4.4214
67.801	1.381	0.1	(4 1 1)	33.9	0.362	4.5496
69.924	1.3442	0.2	(3 3 1)	34.962	0.372	4.6742
72.019	1.3102	2.5	(0 2 4)	36.009	0.3816	4.7957
74.09	1.2786	11.8	(4 2 1)	37.045	0.3911	4.9141
76.139	1.2492	5.6	(3 3 2)	38.069	0.4003	5.0297
80.187	1.196	2.2	(4 2 2)	40.093	0.4181	5.2534
82.191	1.1719	0.1	(4 3 0)	41.095	0.4267	5.3617
84.186	1.1491	0.1	(4 3 1)	42.093	0.4351	5.4679
86.173	1.1276	10	(5 1 1)	43.087	0.4434	5.5721

Table S3. The ICP-OES analysis results.

Samples	Contents of Co (mg L^{-1})	Contents of Ni (mg L^{-1})	Atomic ratio of Ni: Co
$CoSe_2$@NC-I	4.14	0.28	0.068
$CoSe_2$@NC-II	6.62	0.66	0.100
$CoSe_2$@NC-III	4.95	1.66	0.335
(Ni, Co)Se_2@NC	4.15	2.07	0.500

Table S4. The peak area ratios of Ni^{3+}/Ni^{2+} in $Ni_{2p1/2}$ and $Ni_{2p3/2}$ regions.

	Ni 2p $_{3/2}$		Ni 2p $_{1/2}$	
Peak Area	Ni^{2+}	Ni^{3+}	Ni^{2+}	Ni^{3+}
Ni-$CoSe_2$@NC	731.2164	484.484	365.6082	242.2423
(Ni,Co)Se_2@NC	349.3691	496.9838	174.6845	248.4919

Table S5. EXAFS fitting parameters at the Co K-edge for various $CoSe_2$@NC and Ni-$CoSe_2$@NC-II.

Sample	Coordination	CN	R(Å)	$\sigma^2(\times10^{-3}Å^2)$	ΔE(eV)	R-factor
Co foil	Co-Co	12	2.50	--	--	--
Co_2O_3	Co-O	6	2.09	--	--	--
CoO	Co-O	6	2.13	--	--	--
$CoSe_2$@NC	Co-Se	5.0	2.40	6.5	-5.6	0.68%
	Co-Se	1.8	3.81	3.0	10.0	
Ni-$CoSe_2$@NC-II	Co-Se	6.1	2.42	7.2	0.0	0.21%
	Co-Se	2.6	3.82	4.4	10.0	

Table S6. EXAFS fitting parameters at the Co and Ni K-edge for various samples.

Sample	Coordination	CN	R(Å)	$\sigma^2(\times10^{-3}Å^2)$	ΔE(eV)	R-factor
Ni foil	Ni-Ni	12	2.48	--	--	--
NiO	Ni-O	6	2.08	--	--	--
	Ni-Ni	12	2.95	--	--	
Ni-$CoSe_2$@NC-II	Ni-Se	3.8	2.45	4.8	-2.2	1.81%
	Ni-Se	2.8	3.83	4.4	10.0	

Table S7. Normalized contribution ratio (%) of capacitive- and diffusion-controlled capacities at different rates (mV s^{-1}).

Sample	Contribution	0.3	0.5	0.7	0.9	1.0
$CoSe_2$@NC	Diffusion	49.87	45.23	41.22	37.83	34.84
	Capacitive	50.13	54.77	58.78	62.17	65.16
(Ni, Co)Se_2@NC	Diffusion	45.23	28.09	28.09	22.55	19.79
	Capacitive	54.77	71.91	71.91	77.45	80.21
Ni-$CoSe_2$@NC-II	Diffusion	39.28	35.3	32.29	29.12	27.49
	Capacitive	60.27	64.7	67.71	70.88	72.51

Supplementary references

[1] Perdew, J. P.; Burke, K.; Ernzerhof, M, *Phys. Rev. Lett.* **1996**, **77**, 3865−3868.

[2] Kresse, G.; Joubert, D, *Phys. Rev. B* **1999**, 59, 1758-1775; Blöchl, P. E, *Phys. Rev. B* **1994**, 50, 17953−17979.

[3] A. Mahmood, S. Li, Z. Ali, H. Tabassum, B. Zhu, Z. Liang, W. Meng, W. Aftab, W. Guo, H. Zhang, M. Yousaf, S. Gao, R. Zou, Y. Zhao, *Adv. Mater*. **2019**, 31, 1805430.

[4] F. Xu, Y. Zhai, E. Zhang, Q. Liu, G. Jiang, X. Xu, Y. Qiu, X. Liu, H. Wang, S. Kaskel, *Angew. Chem Int. Ed. Engl.* **2020**, 59, 19460.

[5] P. Zhang, Y. Wang, W. Lei, Y. Zou, W. Jiang, Z. Ma, C. Lu, *ACS Appl. Mater. Inter.* **2019**, 11, 24648.

[6] J. Li, N. Zhuang, J. Xie, X. Li, W. Zhuo, H. Wang, J. B. Na, X. Li, Y. Yamauchi, W. Mai, *Adv. Energy Mater.* **2019**, 10, 1903455.

[7] J. Wu, X. Zhang, Z. Li, C. Yang, W. Zhong, W. Li, C. Zhang, N. Yang, Q. Zhang, X. Li, *Adv. Funct. Mater.* **2020**, 30, 2004348.

[8] J. Ruan, F. Mo, Z. Chen, M. Liu, S. Zheng, R. Wu, F. Fang, Y. Song, D. Sun, *Adv. Energy Mater.* **2020**, 10, 1904045.

[9] G. Qin, Y. Liu, F. Liu, X. Sun, L. Hou, B. Liu, C. Yuan, *Adv. Energy Mater.* **2020**, 11, 2003429; X. Chang, X. Zhou, X. Ou, C. S. Lee, J. Zhou, Y. Tang, *Adv. Energy Mater.* **2019**, 9, 1902672.

[10] X. Ou, L. Cao, X. Liang, F. Zheng, H. S. Zheng, X. Yang, J. H. Wang, C. Yang, M. Liu, *ACS Nano* **2019**, 13, 3666.

[11] Y. Li, J. Qian, M. Zhang, S. Wang, Z. Wang, M. Li, Y. Bai, Q. An, H. Xu, F. Wu, L. Mai, C. Wu, *Adv. Mater.* **2020**, 32, 2005802.
